# Expression of growth hormone gene during early development of Siberian sturgeon (*Acipenser baerii*)

**Published:** 2015-12

**Authors:** Zeinab Abdolahnejad, Mohammad Pourkazemi, Majid Reza Khoshkholgh, Mahtab Yarmohammadi

**Affiliations:** 1 Fisheries Department, Faculty of Natural Resources, University of Guilan Sowmeh Sara, Iran; 2 Iranian Fisheries Science Research Institute/Agriculture Research Education Extension, Organization, Tehran, Iran; 3 International Sturgeon Research Institute, Rasht, Iran

**Keywords:** Siberian sturgeon, Growth hormone, mRNA expression, RT-PCR

## Abstract

The mRNA expression of growth hormone (*GH*) gene in early development stages of Siberian sturgeon was investigated using RT-PCR method. Samples were collected from unfertilized eggs up to 50 days post hatched (dph) larvae in 11 different times. Ribosomal protein L6 (RPL6) transcripts were used as the internal standard during quantification of *GH *mRNA expression. The results showed that the *GH *mRNA could be observed in the eyed eggs and even at unfertilized eggs of Siberian sturgeon. The highest amounts of *GH *mRNA were found at 25 and 50 dph larvae, while the lowest levels were detected at 1 and 3 dph larvae stage. These findings suggest that, the *GH *mRNA play a key role during developmental stages of Siberian sturgeon.

## INTRODUCTION

The world sturgeon resources in their native habitats are declined due to over fishing, poaching, pollution and habitat degradation [1]. These anthropogenic impacts resulted in the reduction, or in some cases decimation, of sturgeon stocks worldwide [2]. Aquaculture has been proposed as a source for caviar and meat production as well as a means to conserve wild populations, either through reducing fishing pressures or by providing animals for stock enhancement.

The Siberian sturgeon (*Acipenser baerii*), is one of the most common species being used in aquaculture due to its fast growth rate and caviar production. The Siberian sturgeon presents a considerably fast growth rate from early larval stages throughout its genetics of the growth process in this species has not been understood so far.

Growth hormone (GH) is produced mainly by the anterior pituitary gland in all vertebrates and is responsible for linear growth. The hormone also participates in the regulation of nitrogen, lipid, carbohydrate and mineral metabolism. [3, 4]. The growth hormone in fish plays a major role in regulation of growth, development, physiological process, immune systems, reproduction function as well as the regulation of ionic and osmotic balance. Several studies clearly demonstrated that GH affects different behavioral features including appetite, foraging, aggression and predator avoidance, which in turn has ecological consequences [5-8].

The growth-promoting action of GH is exerted indirectly through inducing hepatic or insulin-like growth factor I (IGF-I) production, but also directly through binding to its own receptors which expressed in several tissues [3, 4]. Several studies have been conducted on the expression of GH gene during early development of various fish species including gilthead sea bream [9], rainbow trout (*Oncorhynchus mykiss*) [10], milkfish (*Chanos chanos*) [11], orange spotted grouper (*Epinephelus coioides*) [12], alligator gar (*Atractosteus spatula*) [13] and Japanese eel (*Anguilla japonica*) [14].

*GH *transcript levels during early development were reported in some sturgeons species including Chinese sturgeon (*Acipenser sinensis*) [15], Beluga (*Huso huso*) and the Persian sturgeon (*Acipenser persicus*) [16]. The objective of the present study was to investigate the expression of *GH *mRNA during early development stages of the Siberian sturgeon in order to better understanding the early life stages of this important fish.

## MATERIALS AND METHODS

**Sampling protocol: **Fertilized and unfertilized eggs of Siberian sturgeon were obtained from fish reproduction center at the International Sturgeon Research Institute, Rasht-Iran in 2012. Spawning was induced by intramuscular injection of LHRH-A2 (3µg per kg body weight) [17]. Eggs were incubated in Zoak incubators (13-14˚C). After 7 days of incubation, the hatched larvae fed with newly hatched *Artemia franciscana *nauplii ad libitum. Specimens were exposed to a 12 L: 12 D photoperiod using overhead fluorescent lights. Water temperature, dissolved oxygen and PH were 14-19 ˚C, 7.5-8.5 ppm and 7-8, respectively. At 10 days posthatch, fish were fed for the first time with a commercial diet for fish larvae at a feeding rate of 15% of total body weight per day. Samples were collected at 11 different stages including unfertilized eggs (UF), eyed eggs (2 days before hatch), newly hatched larvae (0), larvae 1 (transferred from incubators to rearing site), 3, 6, 10 (started of exogenous feeding), 15, 20, 25 and 50 days post-hatching (dph). All fish were killed with overdose of Tricaine Methanesulfonate (MS222). Eggs and larvae samples were washed with DEPC water and rapidly deep-frozen in liquid nitrogen, and kept at -80˚C until RNA was extracted.

**RNA extraction and cDNA synthesis: **Total RNA was extracted from a pool of three sample units (eggs or larvae) using BIOZOL Reagent (Bioflux- Bioer, China) [18]. The RNA quality and quantity was verified using a Nanodrop spectrophotometer (Nanodrop technology, Wilmington, DE, USA) via examination of absorbance ratios at OD 260/280 and by visual inspection of the integrity of both the 18S and 28S ribosomal RNA bands on a 1.5 % agarose gel.

Total RNA was treated by DNAse to remove genomic DNA contaminating. First strand cDNA was reverse-transcribed from 5 µg of total RNA using BioRT cDNA Synthesis Kit (Hangzhou Bioer Technology, Ltd, Hangzhou, China) according to manufacturer’s protocol. The quality and quantity of cDNA was determined using (1%) agarose gel electrophoresis and Nanodrap spectroscopy (ND1000, USA).

The reaction mixtures for PCR amplification were consisted of 3 µl of first strand cDNA, 1.5 µl of dNTPs (10 mM), 0.5 µl of primers each (10 pm), 2 µl MgCl2 (50 mM), 2.5 µl 10X reaction buffer, 0.3 µl Taq polymerase (5U/µl), with sterilized double water added to make up the volume distilled to 25 µl. The PCR condition was as follow: an initial denaturing step at 94˚C for 3 min, 40 cycles of denaturation at 94˚C for 30s, annealing at 61˚C for 30s, extension at 72˚C for 30s and final extension at 61˚C for 5 min. PCR products were electrophoresed on 1.5% agarose gels and visualized using staining with ethidium bromide.

**Real-time PCR: **Real-time PCR analyses were run in triplicate using the CFX96 Real-time PCR system (Bio-Rad) using BioEasy SYBR Green I Real Time PCR Kit (Hangzhou Bioer Technology, Ltd, Hangzhou, China).The specific primers used in RT- PCR were designed according to the conserved regions of the partial sequences of *A. baerii *cDNA-*GH *(GenBank Accession # Fj428829.1) (Table 1). All Primers were designed using Oligo software (version 5.0). PCR condition for all primers was the same, that is, the first holding time at 94˚C for 2 min, followed by a two-step PCR program including: 94˚C for 10s and 60˚C for 30s for 40 cycles. For cDNA normalization, a ribosomal protein L6 (RPL6) was used as housekeeping gene [19] (Table 1). Standard curves were generated for each primer pair using dilution series of pooled cDNA (including four serial dilutions from 1 to 1/1000). The PCR efficiency (E %) was calculated based on [%E = (101/slop -1) ×100] equation as stated at Radonic *et al*. 2004 [20] (Table1).

**Table 1:** Name, sequence, Amplicons, annealing temperature (T) and PCR efficiency (E) of primers, used in the present study

Primer name	Sequence	**T (** **o** **C)**	**Amplicon (bp)**	**E (%)**
RPL6	GTGGTCAAACTCCGCAAGA	60	149	99
	GCCAGTAAGGAGGATGAGGA			
GH	CCATCCCTGCTCCCACTGGC	60	159	98
	ACACTCGGTCGGAGGTGCTG			

**Data analysis: **Relative *GH *mRNA expression was calculated using the 2-ΔΔct method [21]. The calibrator sample was chosen from samples egg, embryo and larval (from 0 to 50 dph) of Siberian sturgeon. Statistical analyses for different comparisons of *GH *mRNA expression during different developmental stages were done using one-way ANOVA and subsequent Tukey's HSD post hoc analysis for multiple comparisons. The level of significant was P<0.05 and the SPSS version 16.0 was used for data analyses.

## RESULTS AND DISCUSSION

Investigation of the RNA bands resolution on the agarose gel showed that the extracted RNA has an acceptable quality. The ratio of absorption intensity in the extracted RNA samples at both wavelengths of 260 and 280 nm were in the range of 1.9-2 which represents the acceptable quantity of extracted RNA from different samples.

**Figure 1:**
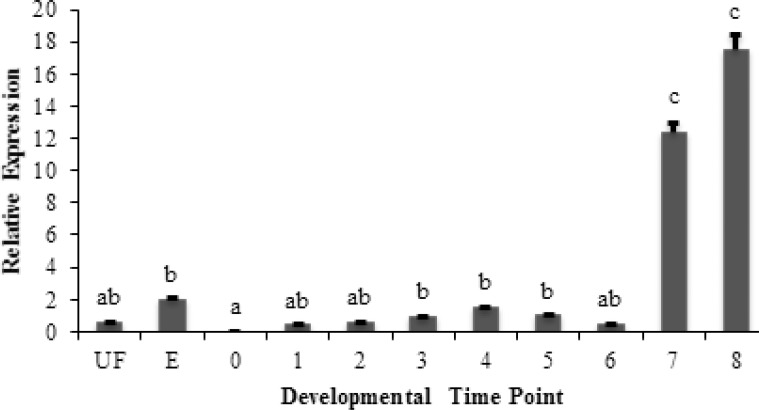
Relative expression levels of *GH* mRNA during early development of Siberian sturgeon. Abbreviations: (UF) unfertilized eggs, (E) eyed eggs, (0) newly hatched larvae, (1) 1dph, (2) 3dph, 3-(6dph), 4-(10dph), (5) 15dph, (6) 20dph, (7) 25dph, (8) 50dph.Total RNA was reverse -transcribed and used for quantitative real-time PCR. The relative amount of the Siberian sturgeon *GH* mRNA was normalized to that level of RPL6 from each RNA sample. Relative *GH* mRNA expression was analyzed by the 2-ΔΔCT method. The data are shown as mean ± SEM; n=6. Statistical significance of differences of the normalized *GH * mRNA data between groups was analyzed using one-way ANOVA and followed by Tukey’s test. Different letters indicate significantly different values at P<0.05.

Standard curves exhibited correlation coefficients higher than 0.98 and the corresponding real-time PCR efficiencies were 0.99 for *GH *and 0.96 for *RPL6*. The changes of GH gene expression during developmental stages of Siberian sturgeon are presented in (Fig. 1). Data were normalized to the housekeeping gene RPL6 and reported relative to 1 dph used as the calibrator sample. The results showed significant differences in expression of *GH *mRNA between the analyzed groups (P<0.05). Also the results of RT-PCR indicated that *GH *mRNA could be detected in the eyed eggs and even unfertilized eggs of Siberian sturgeon. The *GH *mRNA was not found in newly hatched larvae. As depicted in the graph (Fig. 1), the highest amounts of GH mRNA were found in 25 and 50 dph larvae, that showed significant different with other groups (P<0.05).

Real time-PCR showed changes of *GH *gene expression levels during developmental stages of Siberian sturgeon. The GH gene expression was detected in the Siberian sturgeon even in unfertilized eggs indicating that this gene is active in the maternal gametes. Expression of *GH *in oocyte seems to occur in a wide range of vertebrates. In mammals, *GH *mRNA was observed in placentas monkey [22], ovine [23], and human [24]. In fish, similar to mammals, *GH *mRNA was found in mature oocytes in orange spotted grouper [12], *Alligator gar *[13] and Rainbow trout [10], similar to our observations in this study. Nevertheless, it was reported that *GH *mRNA was not found in the ovulated eggs of Japanese eel [14], while the reason of that has not been determined.

In the present study, a significant increase of *GH *mRNA was observed in eyed eggs. In the same direction, the pituitary gland formation before hatching was reported in sturgeons [25]. Furthermore, obvious increase in *GH *mRNA expression levels was observed in embryonic stage of Siberian sturgeon which may be related to the formation of pituitary gland and the endocrine action of pituitary in this fish.

Moreover, in fish larvae, *GH *mRNA expression levels were observed for the first time in the 1 dph Orange-spotted grouper [12], at 2 dph in milkfish [11], at 3 dph in gilthead sea bream [9] and at 6 dph in Japanese eel [14]. In the present study, *GH *mRNA in the larvae of Siberian sturgeon was detected at 1dph for the first time. Our present study suggesting that *GH *is essential for larval growth in the Siberian sturgeon after hatching, as in other fishes. Also these finding suggest that, the pattern of *GH *mRNA expression levels in early developmental stages are not similar in different fish species.

In this research, *GH *mRNA was not detected in newly hatched larvae; however, the reason was not clear. The lowest levels of GH gene expression were observed on days 1 and 3 post-hatching followed by an elevation up to insignificant at 10 dph.

It was reported that during early developmental stages of Siberian sturgeon, the sense organs were poorly differentiated and digestive glands were not developed after hatching [26]. The mouth was opened [27] and the rudiments initial of liver and exocrine pancreas were observed between 1 and 2 dph [26]. It was also reported that the eye started to differentiate at 3 dph [28]; and proteins and lipids of the liver started to accumulate at 3 and 4 dph [26]. Furthermore, the liver and pancreas were differentiated at the same time [26].The development of visual organ was completed in 5-6 dph [28]; and larval food seizure and sense organs and digestive system were completely developed to start exogenous feeding in 9-10 dph, [29, 30]. Our findings suggest that *GH *mRNA possibly plays an essential role in the development of visual organ and onset of exogenous feeding.

Development of Siberian sturgeon's larvae during first week is characterized by organogenesis and fast growth rate. Our results suggest that, the transcription of *GH *is involved in the organogenesis and fast growth rate during early Siberian sturgeon larvae stages.

In the present study, *GH *expression decreased from day 15 to 20 post-hatch. It has been reported that in Siberian sturgeon few morphological changes take place from days 11 to 18 [27]. The Siberian sturgeon growth (in length) was relatively slow in 20 dph larvae [31]. These results suggest that, *GH *is implicated in morphological and morphometric changes during early life stages of Siberian sturgeon.

The expression levels of *GH *mRNA were increased in 25 dph larvae. It was reported that between days 20 and 40 metamorphosis took place and some new juvenile traits appeared. [26]. Furthermore, it was suggested that *GH *may play a key role in the process of metamorphosis during larval stage in grouper [12], and during leptocephalus stage in *Japanese eel *[14]. These findings suggest that *GH *may play an important role during the process of metamorphosis in Siberian sturgeon.

At 50 dph, metamorphosis was completed and specimens resembled miniature adult Siberian sturgeon. The highest level of *GH *gene expression in Siberian sturgeon was detected at 50dph. The results of our study were contradicted with similar observation in different fish species like the Beluga sturgeon (*Huso huso*) and the Persian sturgeon (*Acipenser persicus*) [16]. This difference could be related to variable ontogeny of GH gene among fish species. The increase in *GH *mRNA expression levels in 50 dph larvae of Siberian sturgeon may be explained by the metamorphism completion and beginning of juvenile phase.

In conclusion, our findings demonstrate that the mRNA expression levels of *GH *gene are low during early larval stages followed by a significant increase in the late larval stages. Also, our study shows that *GH *gene is essential for the larvae and embryos growth, suggesting that this gene plays an important role at the early development of Siberian sturgeon.
